# Evolutionary Implications of Metal Binding Features in Different Species’ Prion Protein: An Inorganic Point of View

**DOI:** 10.3390/biom4020546

**Published:** 2014-05-23

**Authors:** Diego La Mendola, Enrico Rizzarelli

**Affiliations:** 1 Department of Pharmacy, University of Pisa, Via Bonanno Pisano 6, Pisa 56126, Italy; 2 Institute of Biostructures and Bioimages, National Council of Research (CNR), Viale A. Doria 6, Catania 95125, Italy; E-Mail: erizzarelli@unict.it

**Keywords:** prion, copper, metal ions, chicken, mammal, peptide, coordination chemistry, neurodegeneration

## Abstract

Prion disorders are a group of fatal neurodegenerative conditions of mammals. The key molecular event in the pathogenesis of such diseases is the conformational conversion of prion protein, PrP^C^, into a misfolded form rich in β-sheet structure, PrP^Sc^, but the detailed mechanistic aspects of prion protein conversion remain enigmatic. There is uncertainty on the precise physiological function of PrP^C^ in healthy individuals. Several evidences support the notion of its role in copper homeostasis. PrP^C^ binds Cu^2+^ mainly through a domain composed by four to five repeats of eight amino acids. In addition to mammals, PrP homologues have also been identified in birds, reptiles, amphibians and fish. The globular domain of protein is retained in the different species, suggesting that the protein carries out an essential common function. However, the comparison of amino acid sequences indicates that prion protein has evolved differently in each vertebrate class. The primary sequences are strongly conserved in each group, but these exhibit a low similarity with those of mammals. The N-terminal domain of different prions shows tandem amino acid repeats with an increasing amount of histidine residues going from amphibians to mammals. The difference in the sequence affects the number of copper binding sites, the affinity and the coordination environment of metal ions, suggesting that the involvement of prion in metal homeostasis may be a specific characteristic of mammalian prion protein. In this review, we describe the similarities and the differences in the metal binding of different species’ prion protein, as revealed by studies carried out on the entire protein and related peptide fragments.

## 1. Introduction

The evolutionary tree of species can be translated, from a chemical point of view, as the chemical evolution of an ecosystem containing classes of organisms in different environments [1].

The basic principles involved in the bioselection of elements are essentially governed by the abundance of the element, its basic fitness for a given task and the evolutionary pressure [1]. In the first stage of life, the reducing atmosphere favored the availability of some chemical elements, such as iron(II), the natural abundance and redox properties of which allowed the chemistry that was suited for life [2]. At the same time, elements, such as copper and zinc, were not available as present in insoluble Cu_2_S and ZnS. The appearance 10^9^ years ago of dioxygen in earth’s atmosphere led to the oxidation of different elements with catastrophic effects for many living organisms [2,3]. The increased concentrations of metal ions in the sea, in particular of soluble copper and zinc, provided the opportunity for organisms to create and manipulate tissues outside cells, so as to bind them together, giving a novel increase in compartments [2]. The development was that of multi-cellular organisms, which used space external to cells to produce connective tissue to hold cells together all within the body. Copper and zinc were critical to the synthesis and functional uses of connective tissues, of new organic messengers and in the elimination of the secondary oxygen radicals [4].

An interesting feature of all cells is that they have a fixed cytoplasmatic content of free metal ions (the free metallome), different from that, always fixed, of extracellular fluids [5]. It is essential that the free and bound levels of metal ions were homeostatically controlled. These controls are dependent upon uptake and rejection mechanisms at various membranes and, then, in the expression of specific metal transporters [4,5].

Metal ions can be broadly classified as either “bio” or “toxicological” metals based on whether they have a functional role or are detrimental to the organism. Transition metals, such as iron, zinc and copper, are present in the brain at concentrations ranging from 100 to 1000 µM [6].

With the increasing complexity of higher multicellular eukaryotes and a more refined organization, the brain evolved to be the first a control system able to produce a rapid automatic response to the environment and, then, also, through the storage of metal ions, to produce memory [6,7]. The control of metal ion content is basic, and a dysfunction in its homeostasis can result in significant neurological abnormalities [8,9,10].

The overall metal content of brain changes with age, which is one of the major risk factors for neurodegenerative diseases. Alterations in the distribution or levels of metal ions with age might be important in the underlying disease pathogenesis [11,12,13].

Among neurodegenerative disorders, the prion diseases are a group of transmissible neurodegenerative disorders affecting many species, including humans (Creutzfeldt-Jakob disease, CJD, and Gerstmann-Straussler-Scheinker disease, GSS), cattle (bovine spongiform encephalopathy, BSE), sheep and goats (scrapie), deer and elk (chronic wasting disease, CWD) [14]. All these diseases are characterized by the accumulation in the brain of an abnormally folded form of the prion protein, which is infectious in the absence of nucleic acid [15,16]. This abnormally folded form of the prion protein is called scrapie prion protein (PrP^Sc^), and it shows more β-sheet and less α-helical content than the normal cellular form, PrP^C^ [17]. The infectious PrP^Sc^ is believed to catalytically convert PrP^C^ to PrP^Sc^ upon contact, thereby spreading the neurotoxic isoform of the protein in brain tissue. Therefore, the key molecular event in the pathogenesis of such diseases is the conformational conversion of PrP^C^ into PrP^Sc^, and subsequent disorders need the presence of PrP^C^, since the absence of endogenous PrP^C^ totally precludes PrP^Sc^-mediated infectivity and neurotoxicity [18]. However, at present, the detailed mechanistic aspects of prion protein conversion remain unclear and, so, the precise physiological function of PrP^C^ in healthy individuals.

PrP^C^ is a highly conserved protein in mammals, but genes encoding homologous prion proteins have been reported in different animal classes, such as avian, reptiles, amphibians and fish, but not in invertebrates [19,20,21,22,23,24]. It is important to point out that prion diseases are observed only in mammals, appearing to be precluded to other species. Thus, the comparison between prion proteins of different species can reveal functional evolutionary trends related to certain aspects of prion’s physiological role and pathogenesis.

In this review, we describe the similarities and the differences in the metal binding of prion protein owing to different species, as revealed by studies carried out on the entire protein and related peptide fragments.

## 2. Prion Protein Biological Role

PrP^C^ has been detected in different tissues, but it is mainly expressed in the brain [25]. It is widely distributed in the neurons and glial cells of the central nervous system (CNS), including olfactory bulb, neocortex, striatum, hippocampus and cerebellar cortex [26]. Not many studies have been carried out on the prion protein distribution in the central nervous system of other animal classes. Among these, the chicken prion protein (chPrP) is the best characterized, and its localization in the CNS has been detected in the dendrites and axons of neurons as a mammalian counterpart [27]. This localization suggests that PrP^C^ may perform a regulatory role in synapse formation and/or the maintenance of a synaptic organization in both avian and mammal classes. However, some regional differences suggest in birds an association of prion with processing sensory information [27].

The specific PrP^C^ physiological role is still an open question. Many studies have suggested a wide range of other different functions, including oxidative stress protection, apoptosis, cellular signaling, cell adhesion and differentiation, angiogenesis and metal ion trafficking [28,29,30,31,32]. The various functions attributed to PrP^C^ suggest its involvement in biochemical pathways that affect several cellular processes.

The exposure to transition metal ions may induce structural changes driving the conformational switch from PrP^C^ to PrP^Sc^ [33,34,35,36], with the induction of toxicity.

Alternatively, the functional role of PrP^C^ may be to regulate copper trafficking and to attend to its metabolism [37,38]. Noteworthy is that within the central nervous system, PrP^C^ is concentrated at presynaptic membranes, a region of high copper localization and flux, suggesting that PrP^C^ may act as a receptor for cellular uptake or the efflux of copper ions by cells [37,38].

Since copper is a redox-active metal, its dyshomeostasis may induce neurotoxicity, due to the generation of free radicals [39,40]; markers of oxidative stress and imbalance of metallostasis have been reported in prion disease-affected brains [41,42]. Brain extracts from PrP-knockout mice have lower copper content than the wild-type [43].

Actually, micromolar concentrations of copper rapidly stimulate the endocytosis of cell-surface mammalian PrP, via clathrin-coated pits, probably through a direct binding of metal to the protein N-terminal domain [44,45]. Interestingly, the first evidence, about copper, prion and endocytosis, has been reported on chicken prion protein [46]. In this context, the copper binding to the N-terminal domain of protein is essential. It has been suggested [46] that the binding of the copper(II) ion to the chPrP^C^ induces a change in conformation, but how this could be a signal for the internalization is unclear.

PrP^C^ expression and the copper bound to it increase the cellular resistance to oxidative stress. The protein may act as a copper chelating agent, when extracellular copper reaches high concentrations peaks (15–300 µM) as, for example, during synaptic transmission and depolarization [47]. Another hypothesis is that the binding of copper to prion could act directly to detoxify oxygen reactive species, performing superoxide dismutase-like activity (SOD-like) [48,49,50]. The SOD-like activity appears to be a general property of PrP^C^, since it is displayed by both mouse and chicken prion proteins [49].

The commonly held opinion is that PrP^C^ exerts its function via different interactions with its diverse regions [51,52,53,54]; analogously, metal complexes with different domains of PrP^C^ are believed to exert different functions [55,56,57,58], indicating that the role of metal ions and their coordination to PrP^C^ is evidently rather complex. Noteworthy is that many studies have been dedicated to understanding copper interaction with mammalian PrP^C^ and less for prion expressed in other species.

## 3. Prion Protein Features

**Figure 1:**
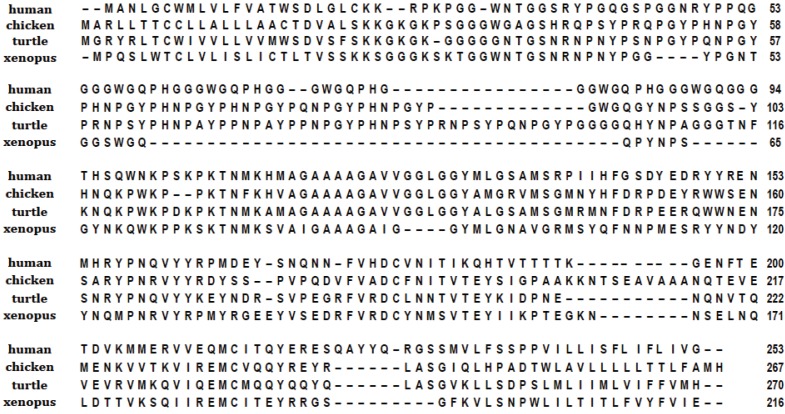
Sequence alignment of different species’ prion proteins.

The comparison among primary sequences suggests that prion proteins have evolved differently in each vertebrate class. Within each group, the sequences are well conserved; strongly in mammal and avian, with similarity scores of 90% [19,20], while in fish, it is lower (50%–60% similarity) [24]. Instead, the sequence similarity between two different classes is rather low, being, on average, about 30% (Figure 1). Prions also exhibit considerable variation in size, ranging from around 260 amino acids in mammals and birds, to the 600 residues in fish [59,60].

**Figure 2:**
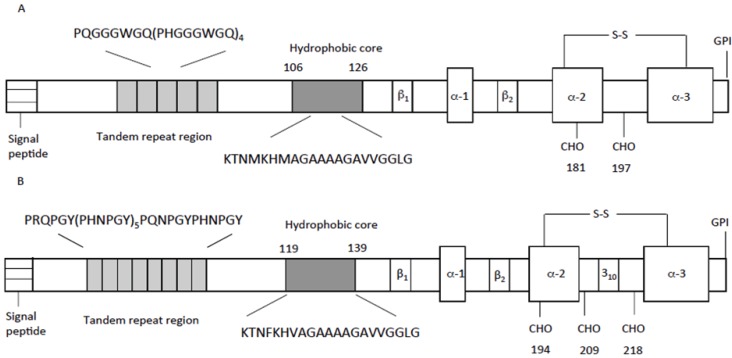
Schematic representation of the domain structure of the (**A**) human prion protein and (**B**) chicken prion protein.

Mammalian PrP^C^ sequences contain a signal peptide (1–22), a highly conserved octapeptide domain (60–91), which contains four repeats with the sequence, PHGGGWGQ, a highly-conserved hydrophobic domain (106–126), three peptide sequences forming an α-helix structure (α1–α2–α3), two peptide sequences forming a β-helix structure and a signal sequence for a glycosylphosphatidylinositol (GPI) anchor (231–253). PrP^C^ contains two consensus sequences for N-linked glycosylation (T181 and T197), and a disulfide bond between Cys 179 and Cys 214 is essential for proper folding of the protein [61,62] (Figure 2A). However, despite the divergence between the primary sequences, the comparison among prions from different species shows the presence of some conserved protein domains [63]. NMR measurements established that chicken, turtle and frog prion proteins show closely similar global folds to that of the mammal one (Figure 2B). The C-terminal domain contains three α-helices, one short 3_10_-helix and a short antiparallel β-sheet, while the N-terminal domain is supposed to be flexible and unordered [63]. The conservation degree of the primary sequence between the vertebrate classes varies significantly along the different protein domain: the short hydrophobic stretch is the most conserved, whereas the N-terminal repetitive region is the most divergent.

The similarity in the globular fold of the C-terminal domain indicates that it has been retained throughout the evolution process, suggesting its relevant biological function [19,20,61,63]. This is to clarify if present prion properties and activities were already present in the last common ancestor of all vertebrates and, then, still common to different animal classes or whether some peculiar properties appeared late in evolution, being a genetic inheritance of higher species, such as mammals.

The NMR comparative analysis of different prion structures through molecular dynamics simulations indicate that in frog, chicken and turtle, the loop between the strand-2 and α-helix 2 is more dynamically disordered and with higher levels of flexibility than that observed in the analogous region of PrP^C^ of mammals [64].

The N-terminal domain encompassing the repeat region has been reported to be flexible in all prion protein of different species [63]. However, differently from mammalian homologues, the digestion of chicken prion protein with trypsin or proteinase K produces peptide fragments stable to further proteolysis encompassing the N-terminal domain [65], suggesting that they adopt a structure different from that of mammalian prion tandem repeats, giving a possible explanation of prion diseases lacking in birds. CD and NMR studies carried out on peptide fragments encompassing the avian repeat domain indicate that these adopt a turn or a PolyPro II (PPII) conformation [66,67,68,69].

The differences found for the secondary structures of chicken and mammalian tandems can be associated with the different ratio between Gly and Pro residues. The mammalian octarepeat peptides contain 50% of glycine and 12% of proline residues, respectively, while the chicken hexarepeats encompass 16% of glycine and 33% of proline residues. The greater flexibility conferred by glycine residues might explain why the mammalian prion protein N-terminal tandem amino acid repeats are unordered. On the contrary, the high number of proline residues can explain the tendency of avian hexarepeats to form turn and/or PPII structures.

The N-terminal domain within each vertebrate class contains a distinctive default number of degenerate repeats that share some amino acids in the basic unit: PHGGGWGQ in mammals; PHNPGY in avian; PxNP in turtle; PxxP in frog and pufferfish; GxxG in zebrafish.

Even though the presence of internal repeats occurs in 14% of all known proteins, this motif in eukaryotic proteins is three times more likely to be present than in prokaryotic ones. It has been proposed that the protein containing repetitive sequences may evolve more quickly, allowing faster adaption to new environments [70]. From fish to human, the repeat units within one molecule have reduced degeneracy, but an increased size, reaching a maximum of eight amino acids in mammals.

The comparison between the sequences of the proteins shows the increase in the number of histidines and the decrease of proline residues in the repeat region, from amphibian to mammalian species. The histidine imidazole side chain represents one of the main copper ion anchoring sites in proteins, whereas the proline residue acts as a break point for copper(II) coordination. These aspects seem to suggest that the evolutionary line moves toward a more effective ability to bind a greater number of Cu^2+^ ions with higher complexation constants.

## 4. Copper(II) Coordination Features within the N-Terminal Domain of Evolutionarily Different Prion Proteins

### 4.1. Mammals

It is widely accepted that the flexible N-terminal domain of mammalian prion protein represents the main binding site of divalent metal ions and, in particular, of copper(II) ions. The tetraoctarepeats (OR) can bind from one up to four copper ions, while a fifth non-OR binding site has been located within the 91–111 sequence, involving the hydrophobic core region [71].

**Figure 3:**
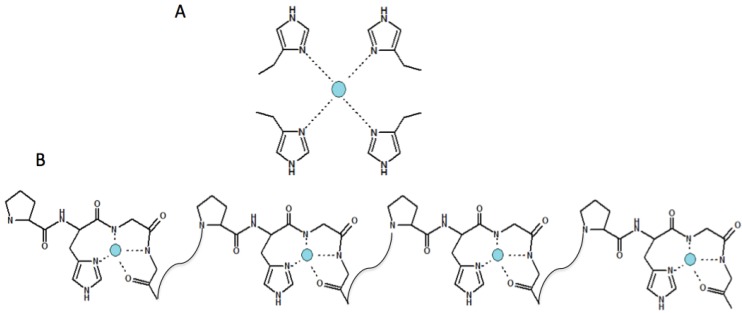
Sketches of different copper coordination modes with the mammal tetra-octarepeat: (**A**) Cu(4N_Im_) low Cu^2+^ occupancy; (**B**) Cu(N_Im_, 2N^−^,O) high Cu^2+^ occupancy.

The mammal histidine-containing octarepeats bind copper(II) at physiological pH with two main coordination modes, depending on the copper/peptide molar ratio [72]. At a sub-stoichiometric copper(II) concentration, the predominant species consists of four imidazole nitrogen atoms bound to the metal ion. Increasing the copper amount, the copper(II) is bound to each octarepeat by means of one imidazole nitrogen, two amide nitrogens and one carbonyl oxygen (see Figure 3B) leading to a [CuLH_2_] species [72,73,74,75,76]. These copper(II) complex species have different K_d_ values, showing a negative cooperative effect [77]. The affinity for the copper decreases as a function of increasing metal ion concentration, starting from a K_d_ of 0.12 nM, when only one copper ion is bound to the tetra-octarepeat, to a K_d_ of 7–12 µM when the same peptide binds four copper ions [77]. These data suggest that the copper-assisted PrP^C^ function can change as a function of extracellular copper levels (Figure 3).

Moreover, the different copper(II) complexes with OR display distinctive electrochemical behaviors [78]. The Cu^2+^ centers in these complexes can be reduced by cellular reductants. This requires the presence of Cu^+^, and it has been reported that the copper(I) complex formed at low occupancy is about three orders of magnitude stronger than that of Cu^2+^ [78,79].

### 4.2. Other Species

Differently from mammalian prion proteins, no results have been reported, as far as we know, for metal interaction with the entire turtle, frog and fish prion protein.

Studies have been focused on copper(II) interaction with peptide fragments owing to the N-terminal domain of pufferfish (*Takifugu rubripes*) and of zebrafish. These Gly-rich peptides form metal complexes at pH = 7.4 in which the copper(II) is bound to two imidazole and two amide nitrogen atoms (2N_Im_, 2N^−^) or form inter-repeat binding, involving three imidazole nitrogen atoms [80,81,82]. The sequence of the fish prion N-terminal domain is more different from that of mammals, whereas analogous avian prion domain shows more similarity.

Conflicting results have been reported as to whether avian PrP^c^ binds copper(II) ions. Marcotte and Eisenberg have shown that copper(II) addition destabilizes the protein, while MALDI-TOF mass spectrometry and CD experiments indicate that there is no specific binding of the metal to the protein [65]. On the contrary, other authors report that both chPrP^c^ and hPrp^c^ bind copper(II) ions, but only when the metal is added during the protein refolding process [49]. The two proteins cannot be compared in detail, since, to the best of our knowledge, no stability constants for the metal ion binding to the whole chicken prion protein have been reported so far.

However, several studies have been conducted on the interaction of copper(II) ions with peptide fragments belonging to the N-terminal domain of the protein.

The first investigation on copper(II) coordination with peptides encompassing the tetra-hexarepeat domain (NPGYPH)_4_ has been performed by means of MALDI-TOF, gel filtration, fluorescence and CD. Data, obtained by using a high metal/peptide molar ratio, suggested that the peptide binds four metal ions [83]. The dissociation constant, calculated by means of fluorescence quenching, gives a value of 4.5 µM that is similar to that obtained for the analogous complex formed with the human tetra-octarepeat (Kd = 6.7 µM) [84]. These conclusions have partially been confuted through CD investigations of copper(II) interaction with the peptide (NPGYPH)_2_NPGYP encompassing two His residues [85]. This study indicates that both the stoichiometry and metal binding mode of chicken hexarepeats are different from those of the mammalian octarepeats, as the chicken bis-hexarepeats can bind only one metal ion per molecule. By contrast, studies carried out with mono-, bis- and tetra-hexarepeats of the sequence, HNPGYP, indicate that each hexarepeat can bind one copper(II) ion with the same copper(II)/peptide stoichiometry observed for the human octarepeat. The stability constants suggest a cooperative effect also for chicken peptide fragments, though the chicken peptide affinity for copper(II) has a lower affinity [86].

These conflicting results can be partially explained by the use of different hexarepeat sequences. The characterization of copper(II) complexes with the peptides, PHNPGY, HNPGYP and NPGYPH, highlights the relevance of the proline residue as a break-point in the metal coordination and the relevance of the histidine residue position in the sequence [87].

**Figure 4:**
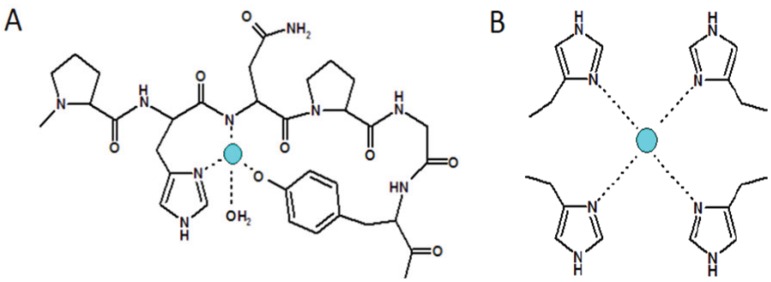
Sketches of different copper coordination modes with (**A**) chicken hexarepeat, Cu (N_Im_, N^−^, O_tyr_^−^); (**B**) Cu (4N_Im_) main species also at low Cu^2+^ occupancy.

As a matter of fact, the stability constants and binding details of copper(II) complexes are not exactly the same, depending on the relative position of histidine and proline residues within the primary sequence. The stability constant value for the metal complex species with HNPGYP, [CuL]^2+^, is larger than that for the analogous species obtained with PHNPGY and NPGYPH [87]. Indeed, PHNPGY and NPGYPH peptides form copper(II) larger chelate rings that are, consequently, less stable than the HNPGYP 7-membered chelate ring. The [CuLH_-1_] complex with HNPGYP is more stable than the two analogous species with PHNPGY and NPGYPH [87]. The [CuLH_-2_] species forms within a narrow pH range, involving tyrosine residues, and in a very low percentage, just before precipitation occurs (Figure 4A). The potentiometric results obtained exploring different metal to ligand molar ratios evidence the propensity of hexarepeat peptide to form bis-complex species [CuL_2_], in which the copper ion is bound to two imidazole nitrogen atoms. An analogous complex species has not been reported for the bis-octarepeat peptide [76,77].

X-ray absorption spectroscopy results for Cu^2+^ complexes with both mammal and avian prion repeats confirm that the chicken tandem hexarepeat as a copper(II) coordination binding mode different from that of the mammalian octarepeat: the avian hexarepeat is more apt to bind copper(II) ions in an inter-repeat than in an intra-repeat mode [88]. In fact, at physiological pH, the peptide encompassing two hexarepeat units, (PHNPGY)_2_, shows a tendency to bind tightly one copper(II) ion through two imidazole nitrogen atoms from two adjacent hexarepeats. Potentiometric measurements showed that the logβ of this species is larger than that of the analogous complex formed by the corresponding bis-octarepeat [76,87].

The (PHNPGY)_2_ can also bind two copper ions in an intra-repeat mode that, however, involves the tyrosine phenolic group, as evidenced by the appearance of a charge transfer band at 390 nm in the UV-Vis spectrum [87]. These binuclear species, [Cu_2_LH_-3_] and [Cu_2_LH_-4_], formed by the bis-hexarepeat, are minor species and are less stable than those formed by bis-octarepeat, coherently with the different donor atoms involved in the bis-hexarepeats (2N_Im_, 2N^−^, 2O^−^) in comparison with those involved in the bis-octarepeats complexes (2N_Im_, 4N^−^), respectively [85,87].

A study on the copper(II) interaction with the chicken tetra-hexarepeat (PHNPGY)_4_ further highlights the difference with the human tetra-octarepeat [89]. The potentiometric and spectroscopic results obtained for the copper(II) complexes with the avian peptide, Ac-(PHNPGY)_4_NH_2_, shows that the species, [CuLH_4_], in which the metal ion is coordinated by four imidazole nitrogen atoms, predominates over a wide range of pH and up to a 2:1 copper/ligand molar ratio. The spin Hamiltonian parameters suggest that copper(II) coordinates via four coplanar equatorial imidazole nitrogen atoms in a pseudo-octahedral geometry, as also confirmed by the UV-Vis spectroscopic results [89] (Figure 4B). The chicken tetra-hexarepeat peptide is able to bind two copper ions only with the direct involvement of tyrosine residues, as confirmed by the presence of the charge transfer band due to deprotonated phenol group. Such a complex species is a minor one, and its coordination mode has to be considered peculiar for avian tetra-hexarepeat peptides, since the analogous mammal tetra-octarepeat does not contain tyrosine residues.

The main differences between the copper(II) complexes formed by chicken hexarepeat and octarepeat arises from the major propensity of the former to form macrochelate and in the absence of metal species with two deprotonated amide nitrogen atoms. This is mainly due to the higher content of proline residue in the chicken repeat sequence. Differently, the main copper(II) complex species formed with the mammal octarepeat domain is a function of the added copper(II) equivalents [76,77].

The different speciation, stoichiometry and binding features coming out from the potentiometric and spectroscopic studies account for the absence of the interaction of the Cu(II)-tetra-hexarepeat with membrane models, differently from that reported for the Cu(II)-tetraoctarepeat system [90]. Environmental factors, copper(II), pH and membrane-mimicking environments play a role in assisting different conformational preferences of chicken tetra-hexarepeats in comparison with the mammalian tetra-octarepeats [90].

Noteworthy is that the chicken tetra-hexarepeat, Ac-(PHNPGY)_4_-NH_2_, and the human tetra-octarepeat, Ac-(PHGGGWGQ)_4_-NH_2_, show constant formation values of the same order of magnitude (10^8^) for the copper complex species in which the metal is coordinated through four imidazole nitrogen atoms [76,89].

This coordination mode recalls that observed for copper(II) in the SOD-1 catalytic site, although in the enzyme, the metal is tightly bound to four imidazole nitrogens with a forced geometry (entactic state) halfway between a tetragonal and a distorted tetrahedron [91]. Instead, the N-terminal region of PrP^C^ is flexible, in particular owing to the mammalian one for the lower content of proline.

It has been established that copper binding to the octarepeat domain is essential for the SOD-like activity of the mammalian PrP^C^, and it has been postulated that the same occurs for the chicken prion protein [49]. The SOD-like activity of the mammalian and chicken protein has been explored by means of nitro-blue tetrazolium (NBT) indirect assays at pH 7.4.

Analogous measurements carried out on copper(II) complex species of both human tetra-octarepeat and chicken tetra-hexarepeat report I_50_ values (I_50_ represents the concentration of the Cu-peptide system that induces 50% of the inhibition of the NBT reduction) of 0.175 and 0.177 µM, respectively; these values are similar to those reported for copper(II) complexes with other histidine-containing peptides, but smaller, by two orders of magnitude, than that shown by the SOD-1 enzyme (I_50_ = 0.0044 µM) [92]. The main copper(II) complex species formed with a peptide encompassing five hexarepeats displays a lower SOD-1 activity, due to the involvement of five imidazole nitrogen atoms in the metal ion coordination environment and, then, to the consequent different coordination geometry [93]. Indeed, the stability constants for the copper(II) complex species of both human and chicken peptides are at least four orders of magnitude smaller than those of the copper-SOD-1 enzyme and this is in contradiction with their possible SOD-like activity *in vivo*, which should display a stronger affinity for the metal ion. Studies carried out on these systems by means of pulse radiolysis, a direct assay, show that the copper(II) complexes with the tetra-octarepeat show a stoichiometric low superoxide scavenging activity, while the tetra-hexarepeat has no activity at all [89]. These last data suggest that both mammal and chicken PrP^C^ protein do not have a direct SOD-like activity, as reported in some papers [94,95].

## 5. Metal Binding Coordination Environment outside the Repeat Domain

Much evidence suggests the presence of a fifth copper(II) binding site outside the octameric region of mammalian PrP^C^ [96,97,98,99,100]. Two histidine residues have been considered the potential anchoring site, namely His96 and His111. Even though contrasting results have been reported, His111 appears to be the most likely site, displaying a high affinity for copper(II). The His111 is included in the neurotoxic region, 106–126, which is considered critical for the PrP^C^→PrP^Sc^ conformational transition [101]. Copper binding to this region has been shown to promote β-sheet formation and to enhance its neurotoxicity [102,103].

A comparison of the thermodynamic stabilities of the peptides encompassing the domain outside the tetra-octarepeat region showed that the former are more effective chelators of the single octarepeat. Such a difference is due to the presence of a prolyl residue in the octarepeat that induces the formation of (7,5,5)-membered chelates, whereas the peptide encompassing His-96 or His111 forms more stable (6,5,5) fused chelate rings [104]. Interestingly, this region is perfectly conserved in avian prion protein.

The comparison between human and chicken peptide fragments showed similar coordination features. The only remarkable difference is due to a slight contribution of the methionine sulfur atom for the species, 3N1O, formed by the human fragment, in which copper is bound to histidine imidazole and two amides of the His and Lys residues [104]. The copper(II) coordination to the chicken peptide induces the same conformational effects observed in the human peptide. The metal ion drives a change from a random coil towards a structured bent conformation, an effect not observed on an analogous scrambled peptide with a different primary sequence [104].

Potentiometric and spectroscopic measurements carried out on peptide fragments encompassing a longer peptide fragment outside the repeat region of the chicken prion suggest that the His-110 closest to the repeat region binds copper ions with a higher stability constant in comparison with the His-124 closest to the hydrophobic stretch [105]. This is the opposite of what is observed for the analogous mammalian peptide.

However, no studies have been carried out, both on the potential neurotoxicity of the chicken 119–139 peptide and on whether this can be affected by metal ion binding, as reported for the mammalian species.

## 6. Conclusions

It is widely accepted that mammalian PrP^C^ protein binds copper specifically [43,71,96]. Copper binding has been shown to induce conformational changes in PrP^C^, as well as to promote its internalization into cells [44,45,46]. Among the different cofactors that bind to PrP^C^, copper has been proven to be able to modulate prion disease pathogenesis [33,34,35], and despite numerous studies, it still remains unclear whether copper ions promote or attenuate the pathological condition. For this reason, the characterization of the copper binding mode with PrP^C^ is significant for a better understanding of its role in prion biology.

The interaction of this protein with metal ions has been studied by using both the entire protein and different peptide fragments, by means of different techniques. However, studies on the whole protein are limited by its scarce solubility at physiological pH.

The numerous Cu^2+^ binding studies of fragments of PrP have mainly focused on two regions within the unstructured N-terminus domain. These studies include the octarepeat region and the amyloidogenic region (non-OR region). The results have been employed to deconvolute the Cu^2+^ binding modes of the full-length prion protein and to correlate the structural features with both the redox cycling and the conformation changes in the presence of metal ions. In this context, this is to highlight that the same binding site can form different species characterized by both different metal-to-ligand ratios and the number of hydrogen atoms (protonated, unprotonated and deprotonated species) depending on the pH values. Underestimating the metal-ligand equilibria of the species actually forming in solution may lead to uncorrected correlations between a metal complex species, its binding mode and potential biological activity.

Prion protein of frog, turtle and chicken displays a globular fold similar to that of mammals [63]. The sequence of N-terminal repeat region presents different amino acid substitutions that may result in being critical for copper(II) binding and, maybe, also for prion conversion. Such differences might account for the absence of prion-like diseases in species outside mammals. On the other hand, the strict correlation of the prion protein with copper(II) could only be a prerogative of mammals and, therefore, its function in the metabolism of the metal to be tied to the evolutionary process.

This hypothesis is enhanced by recent findings on the direct correlation between PrP^C^ and other neurodegenerative diseases. The concept of neurotoxic signaling by PrP^C^ has been extended for its binding of other β-sheet-rich oligomers, suggesting a potential central role for PrP^C^ in the pathogenesis of common neurodegenerative “conformational diseases”, such as Parkinson’s disease, Huntington’s disease and, mainly, Alzheimer’s disease (AD) [106,107]. It has been reported that PrP^C^, bound to membrane, represents the main cellular receptor for the oligomeric β-amyloid species, mediating their toxicity [107,108]. The exact binding site of β-amyloid oligomers is located to the N-terminal domain of PrP^C^, in particular at the region encompassing the residues 95–110 and the basic stretch preceding the tandem repeat region [108].

It should be noted that even though the exact mechanism of neurotoxicity is not known, in all these pathologies, metal ions play a relevant role.

Peptides encompassing the full metal-binding octapeptide-repeats anchored to the surface of lipid vesicles showed that both copper and zinc are able to induce PrP^C^–PrP^C^ interactions, suggesting that prion may be capable of responding to fluctuations of metal levels in brain tissues [109].

Therefore, the understanding of the different copper(II) complex species formed within the repeat N-terminal region may be useful in shedding light on the biological activity of the protein and processes that can lead to its misfolding and, then, to the pathology.

In this regard, it is interesting to note that specific mutations in the C-terminal globular, as well as the N-terminal domain in lower mammals, such as marsupials, may explain their resistance to developing prion infection. The repeat region of opossum PrP^C^ shows a larger number of amino acid substitutions, and the single repeat, PHPGGSNWGQ, forms different copper(II) complex species in comparison to those of the human octarepeat [110].

The coordination features of the octarepeat domain of mammals have been characterized by means of different techniques; in the physiological pH range, the prevailing complex species of the tetraoctarepeat domain is characterized by an inter-repeat binding mode, involving four imidazole nitrogen atoms, at sub-stoichiometric copper(II) to ligand ratios. Indeed, at full site occupancy, the main species is a tetranuclear complex, showing an intrarepeat coordination environment, involving one imidazole and two deprotonated amide nitrogen atoms.

The analogous repeat region of chicken prion has been also fully characterized, and the data obtained show a different stoichiometry, a different coordination binding mode and a different interaction with membranes. The assumption that the chicken prion has the same biological function and activity of the mammalian prion, but to a lesser extent (less SOD-like activity, metal binding properties), is not convincing. Detailed measurements showed that chicken PrP has no SOD-like activity, does not interact with membrane models, and is less affected by copper(II) presence than mammalian prion.

All the data obtained on huPrP60-91, coupled with the different conformational behaviors and the reduced copper(II) binding ability of the avian peptide, support the hypothesis that the N-terminal regions of human and chicken prion proteins are involved in different physiological functions of prions. Such a difference with mammals may result in a wider consideration of reptiles and amphibians, where the complexation of the cupric ion should be even lower, due to the absence or the low number of histidine residues, as well as the high number of proline in the N-terminal domain.
